# Real-world data in late presentation of HIV infection in Suzhou, China: Results from four consecutive cross-sectional surveys, 2017–2020

**DOI:** 10.3389/fpubh.2023.1084840

**Published:** 2023-02-21

**Authors:** Zhihui Xu, Qiang Shen, Di Wang, Zefeng Dong, Weining Han, Runfang Tian, Kai Zhou, Xuerong Ya, Haiyang Hu

**Affiliations:** ^1^Suzhou Municipal Center for Disease Control and Prevention, Suzhou, China; ^2^Jiangsu Provincial Center for Disease Control and Prevention, Nanjing, China

**Keywords:** late presentation, associated factor, real-world data, China, HIV/AIDS

## Abstract

**Objectives:**

This study aimed to examine the real prevalence of late presentation of HIV infection and to identify factors associated with late HIV presentation among patients with newly diagnosed HIV/AIDS in Suzhou, China.

**Methods:**

Patients with newly diagnosed HIV/AIDS who registered in national AIDS surveillance system from 2017 to 2020 were included in this study. Late presentation (LP) of HIV infection was defined as HIV diagnosis with a CD4 count < 350 cells/μL or an AIDS-defining event. Multivariable logistic regression analyses were used to identify factors associated with LP.

**Results:**

A total of 2,300 patients were enrolled. 1,325 were classified as late presenters, showing a high percentage of 57.6% (95% CI: 54.5–60.7%) and a rise (*P* = 0.004) over the four-year period. Patients with newly diagnosed HIV/AIDS who were older than 24 years of age (aOR = 1.549, *P* = 0.001 for 25-39 years; aOR = 2.389, *P* < 0.001 for 40 years and older), were Suzhou registered residents (aOR = 1.259, *P* = 0.026), and were from inpatient and outpatient (aOR = 1.935, *P* < 0.001) were more likely to be late presentation.

**Conclusions:**

This study showed a high percentage and a rise of late presentation of HIV infection among patients with newly diagnosed HIV/AIDS in Suzhou, China, which is a challenge for future prevention and control of AIDS. Targeted measures should be urgently implemented to reduce late HIV diagnosis.

## Introduction

Acquired immune deficiency syndrome (AIDS) is a chronic infectious disease caused by human immunodeficiency virus (HIV). By the end of 2020, there were about 37.7 million people living with HIV worldwide, and 1.5 million people became newly infected with HIV in 2020 (1). 27.5 million people were accessing antiretroviral therapy (ART) as of the end of 2020 (1). AIDS will continue to be a major public health focus for a long time to come. In response to it, the United Nations has proposed the goal of ending the AIDS epidemic by 2030, promising to achieve the “three 95%” by 2030, that is, 95% of people infected with HIV will be diagnosed, 95% of people who are diagnosed will have access to ART, and 95% of people who receive ART will have the virus suppressed (2). In this process, the first 95% is the foundation.

There is increasing evidence that early diagnosis and initiation of effective ART control the HIV-related morbidity and mortality (3, 4). The early use of ART reduces the size of the HIV reservoir and ensures that patients who start ART do so while preserving their immune function (5, 6). On the contrary, late presentation of HIV infection is associated with poor immune reconstitution, ART failure, and a higher mortality risk. Late presenters place a huge burden on the health system: they may have transmitted the virus to others because they are less likely to be aware of their infection and they require more medical resources than people diagnosed early (7).

The late presentation of HIV infection was defined by the European Late Presenter Consensus working group as a patient presenting for care with a CD4 count < 350 cells/μL or presenting with an established AIDS-defining event, regardless of the CD4 cell count (8). This definition aims at a coordinated care and unified management approach to AIDS in order to improve the standard of care for late HIV presenters. Another study proposed a definition of advanced patients as patients who present with a CD4 count < 200 cells/μL and/or have an AIDS-defining event within a month after HIV diagnosis, or patients with a first-reported CD4 count < 350 cells/μL or an AIDS-defining event within a month after diagnosis (9). A consensus definition of late presentation of HIV infection was proposed to ensure consistency and standard of care (10).

However, there is no such standard definition to describe the group of people living with HIV who present late with HIV infection in China. At present, Chinese Center for Disease Control and Prevention (China CDC) still uses the following definition to evaluate late presentation of HIV infection: persons who diagnosed with AIDS patients, or diagnosed with HIV infections and progressing to AIDS patients within the current year. AIDS patient is defined as a person presenting with a CD4 count < 200 cells/μL or presenting with an AIDS-defining event, regardless of the CD4 cell count. This definition of late HIV presentation has an obvious limitation that it can be influenced by ART. Moreover, the percentages of late presenters were underestimated because of the index assessment system. In China, a national study showed the percentage of patients with late HIV presentation ranged from 35.5 to 42.1% from 2010 to 2014 (11). In addition, 70.2% of newly diagnosed patients had late HIV presentation (according to the European consensus definition) in Guangxi Zhuang Autonomous Region from 2012 to 2016 (12). Another study showed that 40.1% of newly diagnosed patients were defined as having advanced HIV disease (AHD, was defined as an initial CD4 count < 200 cells/μL or an AIDS-defining event within a month after HIV diagnosis) in Guangdong Province from May 2018 to June 2019 (13).

Suzhou City, is an economically developed region with more than 13 million people, located in the southeastern part of China. Suzhou belongs to Jiangsu Province and is close to Shanghai. Toward the end of 2021, few finding about late HIV presentation had been reported. Herein, we conducted this four-year cross-sectional survey to examine the status of late presentation and identify factors associated with late HIV presentation among patients with newly diagnosed HIV/AIDS in Suzhou, China.

## Materials and methods

### Study population

All patients with newly diagnosed HIV/AIDS who registered in the AIDS surveillance system of China CDC between January 2017 and December 2020, whose addresses during diagnosis were located in Suzhou city, Jiangsu province, China, were included in this study. Eligible patients also needed to meet the following inclusion criteria: (1) had CD4+ T lymphocyte counts (CD4 counts) during diagnosis, (2) had CD4 counts detected within 3 months after diagnosis, (3) had CD4 counts detected before on antiretroviral therapy (ART). If there were multiple CD4 counts that meet the inclusion criteria, we chose the first into the study.

### Study design

Four consecutive yearly cross-sectional observational study was conducted to examine the prevalence and trends in late presentation of HIV infection, and identify factors associated with late presentation among patients with newly diagnosed HIV/AIDS. Socio-demographic data (including gender, age, occupation, marital status, ethnicity, education level, and registered residence), HIV transmission route, reason for HIV testing, and CD4 count, were collected from the AIDS surveillance system of China CDC. CD4 count testing was conducted in the AIDS confirmatory laboratory of Suzhou Municipal Center for Disease Control and Prevention (Suzhou CDC). Subsequently, local CDC staff entered the CD4 count data of patients into the AIDS surveillance system.

### Definition

In 2009, the European Late Presenter Consensus working group joined efforts to identify a common definition of what is mean by a ‘late-presenting' patient. Two definitions were agreed upon, as follows. Late presentation: persons presenting for care with a CD4 count < 350 cells/μL or presenting with an AIDS-defining event, regardless of the CD4 cell count. Presentation with advanced HIV disease: presenting for care with a CD4 count < 200 cells/μL or presenting with an AIDS-defining event, regardless of the CD4 cell count (8). In our study, late presentation (LP) and non-LP were distinguished according to the consensus definition of late presentation. That was to say, a late presenter was defined as a patient with a CD4 count < 350 cells/μL or with an AIDS-defining event, regardless of the CD4 count.

### Statistical analysis

Related data were downloaded from the AIDS surveillance system. Patients missing CD4 counts that met the inclusion criteria, were disqualified and not included into statistical analyses. According to the distributions of data, the quantitative variables were grouped and part of the qualitative variables were merged. Frequency analyses were used to show the distributions of socio-demographic characteristics and other variables. Chi square tests were used to compare differences of late presentation between subgroups of characteristics. Chi square trend tests were used to compare differences of late presentation between years and observe trends over time. Factors associated with late presentation were first assessed using univariate logistic regression analysis. Variables with *P* < 0.20 were entered into multivariate regression analysis. Multivariable logistic regression analyses were conducted using forward LR method in order to determine the adjusted odds ratios (aORs). All analyses were conduct using SPSS software (version 19.0). *P* < 0.05 were considered statistically significant.

## Results

### Socio-demographic characteristics of study population

A total of 3,096 patients with newly diagnosed HIV/AIDS registered in the AIDS surveillance system of China CDC from 2017 to 2020, whose addresses were located in Suzhou during diagnosis. Among them, 2,300 eligible patients (513, 627, 583, and 577 in 2017, 2018, 2019, and 2020, respectively) were enrolled into the study. There were no statistically significant differences between their socio-demographic characteristics and those of patients who did not meet the inclusion criteria. Their socio-demographic characteristics are summarized in Table 1. The average age of study population was 38.8 with a standard deviation of 14.7 (range: 2–84). Patients aged 24 years or younger during diagnosis constituted 15.0% (346), those aged 25–39 years constituted 45.7% (1,051), and those aged 40 years or older constituted 39.3% (903). Majority of patients were male (89.2%, 2,051) and ethnic Han (96.7%, 2,225). Approximately half (48.7%, 1,120) had an education level of junior high school or lower. About one-third (33.1%, 761) were Suzhou registered residents and two-third (66.9%, 1,539) were migrant population (Table 1).

**Table 1:** Socio-demographic characteristics and late presentation among patients with newly diagnosed HIV/AIDS.

**Characteristics**	***N* (%)**	**LP Cases**	**Rate (%) (95% CI)**	***P*-value**
**Gender**				0.305
Male	2,051 (89.2)	1,174	57.2 (54.0–60.5)	
Female	249 (10.8)	151	60.6 (51.0–70.3)	
**Age (years)**				<0.001
≤ 24	346 (15.0)	145	41.9 (35.1–48.7)	
25–39	1,051 (45.7)	565	53.8 (49.3–58.2)	
≥40	903 (39.3)	615	68.1 (62.7–73.5)	
**Occupation**				<0.001
Service personnel	768 (33.4)	387	50.4 (45.4–55.4)	
Factory worker	529 (23.0)	307	58.0 (51.5–64.5)	
Homemaker or unemployment	392 (17.0)	248	63.3 (55.4–71.1)	
Retiree	114 (5.0)	80	70.2 (54.8–85.6)	
Farmer	219 (9.5)	155	70.8 (59.6–81.9)	
Others	278 (12.1)	148	53.2 (44.7–61.8)	
**Marital status**				<0.001
Single	1,005 (43.7)	497	49.5 (45.1–53.8)	
Married	943 (41.0)	612	64.9 (59.8–70.0)	
Divorced or widowed	352 (15.3)	216	61.4 (53.2–69.5)	
**Ethnicity**				0.318
Han	2,225 (96.7)	1,286	57.8 (54.6–61.0)	
Ethnic minorities	75 (3.3)	39	52.0 (35.7–68.3)	
**Education level**				<0.001
Junior high school or lower	1,120 (48.7)	689	61.5 (56.9–66.1)	
Senior high school	526 (22.9)	303	57.6 (51.1–64.1)	
College or higher	654 (28.4)	333	50.9 (45.4–56.4)	
**Registered residence**				<0.001
Suzhou	761 (33.1)	506	66.5 (60.7–72.3)	
Others	1,539 (66.9)	819	53.2 (49.6–56.9)	
**Route of transmission**				<0.001
MSM	1,410 (61.3)	756	53.6 (49.8–57.4)	
Heterosexual	880 (38.3)	562	63.9 (58.6–69.1)	
Others	10 (0.4)	7	70.0 (18.1–100.0)	
**Reason for HIV testing**				<0.001
VCT	625 (27.2)	314	50.2 (44.7–55.8)	
Inpatient and outpatient	560 (24.3)	394	70.4 (63.4–77.3)	
Pre-surgery	287 (12.5)	176	61.3 (52.3–70.4)	
STD clinic	280 (12.2)	162	57.9 (48.9–66.8)	
Investigation	363 (15.8)	177	48.8 (41.6–55.9)	
Others	185 (8.0)	102	55.1 (44.4–65.8)	

LP, Late presentation; CI, Confidence interval; MSM, Men who have sex with men; VCT, HIV voluntary counseling and testing; STD, Sexually transmitted disease.

### HIV routes of transmission and reasons for HIV testing

Of the 2,300 patients, 61.3% (1,410) were MSM and 38.3% (880) were infected with HIV through heterosexual sex. Patients were tested for HIV for all different kinds of reasons, and then were found to be infected with HIV. Of those, 49.0% (1,127) were from medical institutions [inpatient, outpatient, pre-surgery, and STD (sexually transmitted disease) clinic], and 43.0% (988) from VCT (HIV voluntary counseling and testing) clinics and investigations (Table 1).

### Late presentation of HIV infection

The overall median CD4 count was 317 cells/μL [inter-quartile range (IQR): 206–441]. Among 2,300 patients, 1,325 were classified as late presenters according to the definition, showing a high percentage of 57.6% (95% CI: 54.5–60.7%). Of those, higher percentages of patients with late presentation were found in those aged 40 years or older (68.1%), retirees (70.2%), farmers (70.8%), married (64.9%), heterosexuals (63.9%), those with education level of junior high school or lower (61.5%), being registered residence of Suzhou (66.5%), and being from inpatient and outpatient (70.4%) (Table 1).

### Trends in late presentation of HIV infection

Over the four-year period, a rise of late presentation of HIV infection was shown (*P* = 0.004), with 52.8% in 2017, 56.8% in 2018, 59.2% in 2019, and 61.2% in 2020, respectively. Increases of late presentation were noted in the patients who were male (*P* = 0.003), 25–39 years (*P* = 0.049), retirees (*P* = 0.039), married (*P* = 0.001), had an education level of junior high school or lower (*P* = 0.046), migrant population (*P* = 0.007), MSM (*P* = 0.009), and were from VCT clinics (*P* = 0.026) (Table 2).

**Table 2:** Trends in late presentation of HIV infection among patients with newly diagnosed HIV/AIDS.

**Characteristics**	**2017**		**2018**		**2019**		**2020**		**Trend test, *P*-value**
	***N***	**LP cases (Rate, %)**	***N***	**LP cases (Rate, %)**	**N**	**LP cases (Rate, %)**	***N***	**LP cases (Rate, %)**	
**Total**	513	271 (52.8)	627	356 (56.8)	583	345 (59.2)	577	353 (61.2)	0.004
**Gender**									
Male	453	234 (51.7)	557	315 (56.6)	519	308 (59.3)	522	317 (60.7)	0.003
Female	60	37 (61.7)	70	41 (58.6)	64	37 (57.8)	55	36 (65.5)	0.732
**Age (years)**									
≤ 24	69	21 (30.4)	94	41 (43.6)	95	44 (46.3)	88	39 (44.3)	0.097
25-39	270	138 (51.1)	283	140 (49.5)	238	138 (58.0)	260	149 (57.3)	0.049
≥40	174	112 (64.4)	250	175 (70.0)	250	163 (65.2)	229	165 (72.1)	0.246
**Occupation**									
Service personnel	200	96 (48.0)	223	104 (46.6)	184	100 (54.3)	161	87 (54.0)	0.114
Factory worker	106	52 (49.1)	143	88 (61.5)	133	78 (58.6)	147	89 (60.5)	0.154
Homemaker or unemployment	84	51 (60.7)	118	77 (65.3)	91	60 (65.9)	99	60 (60.6)	0.947
Retiree	15	10 (66.7)	25	14 (56.0)	30	19 (63.3)	44	37 (84.1)	0.039
Farmer	47	34 (72.3)	53	39 (73.6)	77	53 (68.8)	42	29 (69.0)	0.596
Others	61	28 (45.9)	65	34 (52.3)	68	35 (51.5)	84	51 (60.7)	0.093
**Marital status**									
Single	228	105 (46.1)	262	122 (46.6)	249	127 (51.0)	266	143 (53.8)	0.050
Married	214	121 (56.5)	267	173 (64.8)	246	161 (65.4)	216	157 (72.7)	0.001
Divorced or widowed	71	45 (63.4)	98	61 (62.2)	88	57 (64.8)	95	53 (55.8)	0.365
**Ethnicity**									
Han	496	263 (53.0)	597	339 (56.8)	572	340 (59.4)	560	344 (61.4)	0.004
Ethnic minorities	17	8 (47.1)	30	17 (56.7)	11	5 (45.5)	17	9 (52.9)	0.925
**Education level**									
Junior high school or lower	230	129 (56.1)	285	174 (61.1)	306	193 (63.1)	299	193 (64.5)	0.046
Senior high school	128	64 (50.0)	153	93 (60.8)	115	68 (59.1)	130	78 (60.0)	0.159
College or higher	155	78 (50.3)	189	89 (47.1)	162	84 (51.9)	148	82 (55.4)	0.257
**Registered residence**									
Suzhou	174	112 (64.4)	197	130 (66.0)	209	134 (64.1)	181	130 (71.8)	0.203
Others	339	159 (46.9)	430	226 (52.6)	374	211 (56.4)	396	223 (56.3)	0.007
**Route of transmission**									
MSM	307	144 (46.9)	387	204 (52.7)	368	213 (57.9)	348	195 (56.0)	0.009
Heterosexual	203	125 (61.6)	238	152 (63.9)	213	130 (61.0)	226	155 (68.6)	0.206
Others	3	2 (66.7)	2	0 (0.00)	2	2 (100.00)	3	3 (100.00)	-
**Reason for HIV testing**									
VCT	143	63 (44.1)	183	88 (48.1)	140	74 (52.9)	159	89 (56.0)	0.026
Inpatient and outpatient	125	82 (65.6)	150	113 (75.3)	137	97 (70.8)	148	102 (68.9)	0.847
Pre-surgery	57	32 (56.1)	83	55 (66.3)	80	44 (55.0)	67	45 (67.2)	0.512
STD clinic	43	21 (48.8)	77	41 (53.2)	75	45 (60.0)	85	55 (64.7)	0.050
Investigation	90	45 (50.0)	89	41 (46.1)	108	56 (51.9)	76	35 (46.1)	0.854
Others	55	28 (50.9)	45	18 (40.0)	43	29 (67.4)	42	27 (64.3)	0.047

MSM, Men who have sex with men; VCT, HIV voluntary counseling and testing; STD, Sexually transmitted disease.

### Factors associated with late presentation of HIV infection

The potential factors associated with late presentation of HIV infection were analyzed with not late presenters as control. In the univariate analysis, late presentation was significantly associated with age, occupation, marital status, education level, registered residence, route of HIV transmission, and reason for HIV testing. In the multivariate analysis, the patients with newly diagnosed HIV/AIDS who were older than 24 years of age (aOR = 1.549, 95% CI: 1.209–1.985, *P* = 0.001 for 25–39 years; aOR = 2.389, 95% CI: 1.815–3.145, *P* < 0.001 for 40 years and older), were Suzhou registered residents (aOR = 1.259, 95% CI: 1.028–1.543, *P* = 0.026), and were from inpatient and outpatient (aOR = 1.935, 95% CI: 1.510–2.479, *P* < 0.001) were more likely to be late presentation (Table 3).

**Table 3:** Univariate and multivariate analysis of factors associated with late presentation of HIV infection.

**Variable**	**LP (%)**	**Non-LP (%)**	**OR (95% CI)**	***P-*value**	**aOR (95% CI)**	***P-*value**
**Gender**						
Male	1,174 (88.6)	877 (89.9)	1.000			
Female	151 (11.4)	98 (10.1)	1.151 (0.880–1.506)	0.305		
**Age (years)**						
≤ 24	145 (10.9)	201 (20.6)	1.000		1.000	
25–39	565 (42.6)	486 (49.8)	1.612 (1.261–2.060)	<0.001	1.549 (1.209–1.985)	0.001
≥40	615 (46.4)	288 (29.5)	2.960 (2.293–3.821)	<0.001	2.389 (1.815–3.145)	<0.001
**Occupation**						
Service personnel	387 (29.2)	381 (39.1)	1.000			
Factory worker	307 (23.2)	222 (22.8)	1.361 (1.089–1.702)	0.007		
Homemaker or unemployment	248 (18.7)	144 (14.8)	1.696 (1.321–2.176)	<0.001		
Retiree	80 (6.0)	34 (3.5)	2.316 (1.514–3.545)	<0.001		
Farmer	155 (11.7)	64 (6.6)	2.384 (1.725–3.296)	<0.001		
Others	148 (11.2)	130 (13.3)	1.121 (0.852–1.475)	0.416		
**Marital status**						
Single	497 (37.5)	508 (52.1)	1.000			
Married	612 (46.2)	331 (33.9)	1.890 (1.575–2.267)	<0.001		
Divorced or widowed	216 (16.3)	136 (13.9)	1.623 (1.267–2.080)	<0.001		
**Ethnicity**						
Han	1,286 (97.1)	939 (96.3)	1.000			
Ethnic minorities	39 (2.9)	36 (3.7)	0.791 (0.499–1.254)	0.319		
**Education level**						
Junior high school or lower	689 (52.0)	431 (44.2)	1.000			
Senior high school	303 (22.9)	223 (22.9)	0.850 (0.688–1.049)	0.130		
College or higher	333 (25.1)	321 (32.9)	0.649 (0.534–0.789)	<0.001		
**Registered residence**						
Suzhou	506 (38.2)	255 (26.2)	1.744 (1.456–2.090)	<0.001	1.259 (1.028–1.543)	0.026
Others	819 (61.8)	720 (73.8)	1.000		1.000	
**Route of transmission**						
MSM	756 (57.1)	654 (67.1)	1.000			
Heterosexual	562 (42.4)	318 (32.6)	1.529 (1.286–1.817)	<0.001		
Others	7 (0.5)	3 (0.3)	2.019 (0.520–7.837)	0.310		
**Reason for HIV testing**						
VCT	314 (23.7)	311 (31.9)	1.000		1.000	
Inpatient and outpatient	394 (29.7)	166 (17.0)	2.351 (1.850–2.988)	<0.001	1.935 (1.510–2.479)	<0.001
Pre-surgery	176 (13.3)	111 (11.4)	1.570 (1.181–2.088)	0.002	1.174 (0.870–1.582)	0.294
STD clinic	162 (12.2)	118 (12.1)	1.360 (1.023–1.807)	0.034	1.324 (0.992–1.766)	0.057
Investigation	177 (13.4)	186 (19.1)	0.943 (0.728–1.221)	0.654	0.972 (0.748–1.264)	0.835
Others	102 (7.7)	83 (8.5)	1.217 (0.876–1.692)	0.242	1.042 (0.742–1.463)	0.813

LP, Late presentation; CI, Confidence interval; MSM, Men who have sex with men; VCT, HIV voluntary counseling and testing; STD, Sexually transmitted disease.

## Discussion

Our study is among the first to examine trends in late presentation of HIV infection over a period of four consecutive years in Eastern China. A slight rise of late presentation of HIV infection for patients with newly diagnosed HIV/AIDS was found in Suzhou, from 2017 to 2020. It is different from most studies. In recent years, there had been decreasing trends among late presenters in Turkey (14), Kinshasa of DRC (15), and the East of England (16). In addition, trends in late presentation of HIV infection remained relatively stable in Australia (17), Panama (18), and Guangxi of China (12). Over the past dozen years, several HIV testing promotion measures and strategies have been widely used in medical institutions in China. For example, patients requiring surgery or transfusion have to receive HIV testing. Some inpatients, dental patients and endoscopy patients are also required to receive HIV testing. The expanded HIV testing strategy has resulted in a large number of late-stage HIV/AIDS being detected. This may be one reason for the rise of late presentation of HIV infection. Another possible explanation could be that HIV prevention and testing efforts in some population had been weakened or neglected, such as retirees, married, migrant population and people with low education.

In our study, high percentages of late presentation were shown over the time, with more than half each year. It is higher than that in some countries of Europe (14, 19–22), Australia (17), and the United States (23), lower than that in Panama (18), South Africa (24, 25), Ethiopia (26), and Guangxi of China (12), and similar to that in Iran (27). It is important to notice that higher percentages of late presentation were indicated among people over 40 years old, retirees, farmers, married, heterosexuals, and people registered in Suzhou in our study. Targeted interventions addressed to specific subgroups in the population are needed.

Late presentation of HIV infection is associated with an increase in AIDS and AIDS-related deaths, particularly in the first year after diagnosis. Reducing late presentation of HIV infection is considered a public health priority and continues to be a great challenge (28). It is clear that late presentation is significantly associated with lack of recent testing. Therefore, earlier testing for HIV is considered the best measure. Given the priority of the Chinese government's response to HIV epidemic among men who have sex with men (MSM) (e.g., expanding HIV testing, Web-based HIV testing, increasing coverage of care and treatment), our observed percentage of late presentation among MSM was lower than that among heterosexuals (29). It indicates that current HIV testing strategies have not been effectively reaching certain segments of population that need to be tested or tested more frequently. HIV testing coverage needs to be expanded further. In order to achieve this goal, the US CDC and the US Preventive Services Task Force recommended that clinicians screen adolescents and adults aged 15 to 65 years for HIV infection (30).

Consistent with most studies (12, 14, 15, 20, 21, 27), age was significantly associated with late presentation of HIV infection in this study. Late presenters were more elderly. This might be due to several reasons. Because of the particularity of HIV infection, HIV symptoms in old people were misjudged as other illnesses, or the elderly were ignored and hard to receive targeted HIV prevention efforts, such as testing. In some areas of China, HIV testing has been incorporated into the physical examination of the elderly in basic public health services. It was reported that, during the year preceding the diagnosis, nearly half of the patients had sought medical advice owing to the presence of clinical indicators that should have led to HIV testing. Of those, 15% were classified as missed opportunities for earlier HIV diagnosis because testing was not performed (31). Besides, psychological factors, such as stigma, are more common in older people, which may also impede access to health services including HIV testing. Different from some studies (12, 18, 27), we did not observe any impact of gender.

In some studies (17, 32), there may be higher late presentation of HIV infection among migrant people compared with permanent residents. On the contrary, the patients with newly diagnosed HIV/AIDS who were Suzhou registered residents, were more likely to be late presentation in our study. The possible reason was the same as that of increasing late presentation. Due to more medical resources for registered residents, more patients with newly diagnosed HIV/AIDS were continuously passively found through surgery, hospitalization, outpatient service, endoscopy, and so on. The patients with newly diagnosed HIV/AIDS who were from hospital were more likely to be late presentation compared with those from VCT clinics and investigations, because of passive testing. Hence, WHO consolidated guidelines on HIV testing services recommended demand creation of HIV testing services. Demand creation was defined as activities intended to improve an individual's knowledge and attitude, motivation and intention, and eventually decision and behavior to seek HIV testing services (33).

Through this study, we obtained real data in late presentation of HIV infection and possible factors associated with late HIV presentation among patients with newly diagnosed HIV/AIDS in Suzhou, China, which are very important for future HIV prevention and control, especially in reducing AIDS-related deaths. However, this study had several limitations. First, the details of the category of AIDS-defining event were unavailable among late presenters based on AIDS-defining event. Second, the patients of this study were from one city, thus leading to a selection bias. Still, the sample is representative because Suzhou has the largest number of patients in the province. Third, a limited number of variables were included in our study, as no additional questionnaire was conducted. Further study will focus on the knowledge of AIDS, attitude toward AIDS, the clinical symptoms and underlying diseases of patients.

## Conclusions

This study showed a high percentage and a rise of late presentation of HIV infection among patients with newly diagnosed HIV/AIDS in Suzhou, China, from 2017 to 2020, which is a challenge for future prevention and control of AIDS. Targeted measures should be urgently implemented to reduce late HIV diagnosis.

## Data availability statement

The raw data supporting the conclusions of this article will be made available by the authors, without undue reservation.

## Ethics statement

Ethical review and approval was not required for the study on human participants in accordance with the local legislation and institutional requirements. Written informed consent to participate in this study was provided by the participants' legal guardian/next of kin.

## Author contributions

HH, XY, and ZX contributed to conception and design of the study. HH, XY, KZ, and ZD organized the database. HH and ZX performed the statistical analysis. QS, DW, WH, and RT conducted laboratory testing. HH wrote the first draft of the manuscript. XY, ZX, and QS wrote the sections of the manuscript. All authors contributed to manuscript revision, read, and approved the submitted version.

## References

[1] UNAIDS. UNAIDS Data 2021. Geneva, Switzerland. (2021) Available online at: https://www.unaids.org/sites/default/files/media_asset/JC3032_AIDS_Data_book_2021_En.pdf (accessed August 5, 2022).

[2] UNAIDS. New Global Pledge to End All Inequalities Faced by Communities and People Affected by HIV Towards Ending AIDS. (2021) Available online at: https://www.unaids.org/en/resources/presscentre/pressreleaseandstatementarchive/2021/june/20210608_hlm-opens (accessed August 5, 2022).

[3] Jensen-Fangel S Pedersen L Pedersen C Larsen CS Tauris P Møller A. Low mortality in HIV-infected patients starting highly active antiretroviral therapy: a comparison with the general population. AIDS. (2004) 18:89–97. 10.1097/00002030-200401020-00011 15090834

[4] Ormaasen V Sandvik L Dudman SG Bruun JN. HIV related and non-HIV related mortality before and after the introduction of highly active antiretroviral therapy (HAART) in Norway compared to the general population. Scand J Infect Dis. (2007) 39:51–7. 10.1080/00365540600904779 17366013

[5] Jain V Hartogensis W Bacchetti P Hunt PW Hatano H Sinclair E. Antiretroviral therapy initiated within 6 months of HIV infection is associated with lower T-cell activation and smaller HIV reservoir size. J Infect Dis. (2013) 208:1202–11. 10.1093/infdis/jit311 23852127 PMC3778965

[6] Chéret A Bacchus-Souffan C Avettand-Fenoël V Mélard A Nembot G Blanc C. Combined ART started during acute HIV infection protects central memory CD4+ T cells and can induce remission. J Antimicrob Chemother. (2015) 70:2108–20. 10.1093/jac/dkv084 25900157

[7] Antinori A Johnson M Moreno S Yazdanpanah Y Rockstroh JK. Report of a European working group on late presentation with hiv infection: recommendations and regional variation. Antivir Ther. (2010) 15:31–5. 10.3851/IMP1525 20442459

[8] Antinori A Coenen T Costagiola D Dedes N Ellefson M Gatell J. European late presenter consensus working group. Late presentation of HIV infection: a consensus definition. HIV Med. (2011) 12:61–4. 10.1111/j.1468-1293.2010.00857.x 20561080

[9] Jiang H Yin J Fan Y Liu J Zhang Z Liu L. Gender difference in advanced HIV disease and late presentation according to European consensus definitions. Sci Rep. (2015) 5:14543. 10.1038/srep14543 26412578 PMC4585954

[10] Kozak M Zinski A Leeper C Willig JH Mugavero MJ. Late diagnosis, delayed presentation and late presentation in HIV: proposed definitions, methodological considerations and health implications. Antivir Ther. (2013) 18:17–23. 10.3851/IMP2534 23341432

[11] Jin X Xiong R Wang LY Mao YR. Analysis on the ' late diagnosis' (LD) phenomena among newly identified HIV/AIDS cases in China, 2010-2014. Zhonghua Liu Xing Bing Xue Za Zhi. (2016) 37:218–21. 10.3760/cma.j.issn.0254-6450.2016.02.014 26917519

[12] Hu X Liang B Zhou C Jiang J Huang J Ning C. late presentation and advanced HIV disease among patients with newly diagnosed HIV/AIDS in Southwestern China: a large-scale cross-sectional study. AIDS Res Ther. (2019) 16:6. 10.1186/s12981-019-0221-7 30876476 PMC6420760

[13] Jiang H Liu J Tan Z Fu X Xie Y Lin K. Prevalence of and factors associated with advanced HIV disease among newly diagnosed people living with HIV in Guangdong Province, China. J Int AIDS Soc. (2020) 23:e25642. 10.1002/jia2.25642 33225623 PMC7680922

[14] Karaosmanoglu HK Mete B Gündüz A Aydin ÖA Sargin F Sevgi DY. Late presentation among patients with human immunodeficiency virus infection in Turkey. Cent Eur J Public Health. (2019) 27:229–34. 10.21101/cejph.a5416 31580559

[15] Ngongo NM Nani-Tuma HS Mambimbi MM Mashi ML Izizag BB Ndolumingu FK. Decrease in late presentation for HIV care in Kinshasa, DRC, 2006-2020. AIDS Res Ther. (2021) 18:41. 10.1186/s12981-021-00366-8 34271957 PMC8283988

[16] Bath RE Emmett L Verlander NQ Reacher M. Risk factors for late HIV diagnosis in the East of England: evidence from national surveillance data and policy implications. Int J STD AIDS. (2019) 30:37–44. 10.1177/0956462418793327 30170527

[17] Marukutira T Gunaratnam P Douglass C Jamil MS McGregor S Guy R. Trends in late and advanced HIV diagnoses among migrants in Australia; implications for progress on fast-track targets: a retrospective observational study. Medicine. (2020) 99:e19289. 10.1097/MD.0000000000019289 32080144 PMC7034696

[18] Robles MA Ortiz AY Zaldivar Y Castillo J Gondola J Mewa JC. Evolution of late presentation to care and advanced HIV in newly HIV diagnosed subjects in the Republic of Panama: 2012-2017. Int J STD AIDS. (2020) 31:791–9. 10.1177/0956462419890761 32487001

[19] Late Presentation Working Groups in EuroSIDA and COHERE. Estimating the burden of HIV late presentation and its attributable morbidity and mortality across Europe 2010-2016. BMC Infect Dis. (2020) 20:728. 10.1186/s12879-020-05261-7 33028235 PMC7541282

[20] Rava M Domínguez-Domínguez L Bisbal O López-Cortés LF Busca C Antela A. Cohort of the Spanish HIV/AIDS research network (CoRIS). Late presentation for HIV remains a major health issue in Spain: results from a multicenter cohort study, 2004-2018. PLoS ONE. (2021) 16:e0249864. 10.1371/journal.pone.0249864 33882093 PMC8059864

[21] Jabłonowska E Szetela B Bielecki M Horban A Bociaga-Jasik M Mularska E. Acquired immune deficiency syndrome (AIDS) and late presentation in Poland - data from test and keep in care (TAK) Polska project. HIV Med. (2021) 22:387–96. 10.1111/hiv.13041 33410278

[22] Miranda AC Miranda M Pingarilho M Pimentel V Torres J Peres S. Determinants of HIV-1 late presentation in a cohort of Portuguese HIV-1 patients. AIDS Res Hum Retroviruses. (2021) 37:846–51. 10.1089/aid.2020.0175 33461392

[23] Nduaguba SO Ford KH Wilson JP Lawson KA Cook RL. Identifying subgroups within at-risk populations that drive late HIV diagnosis in a Southern U. S state. Int J STD AIDS. (2021) 32:162–9. 10.1177/0956462420947567 33327899 PMC7879228

[24] Sogbanmu OO Goon DT Obi LC Iweriebor BC Nwodo UN Ajayi AI. Socio-demographic and clinical determinants of late presentation among patients newly diagnosed with HIV in the Eastern Cape, South Africa. Medicine. (2019) 98:e14664. 10.1097/MD.0000000000014664 30813211 PMC6408115

[25] Fomundam HN Tesfay AR Mushipe SA Mosina MB Boshielo CT Nyambi HT. Prevalence and predictors of late presentation for HIV care in South Africa. S Afr Med J. (2017) 107:1058–64. 10.7196/SAMJ.2017.v107i12.12358 29262956 PMC5792655

[26] Gesesew HA Ward P Woldemichael K Mwanri L. Late presentation for HIV care in Southwest Ethiopia in 2003-2015: prevalence, trend, outcomes and risk factors. BMC Infect Dis. (2018) 18:59. 10.1186/s12879-018-2971-6 29378523 PMC5789710

[27] Mohammadi Y Mirzaei M Shirmohammadi-Khorram N Farhadian M. Identifying risk factors for late HIV diagnosis and survival analysis of people living with HIV/AIDS in Iran (1987-2016). BMC Infect Dis. (2021) 21:390. 10.1186/s12879-021-06100-z 33906638 PMC8077959

[28] Chadwick DR Freedman A. Treating late HIV diagnosis as a patient safety issue in the UK. Lancet HIV. (2019) 6:e346–8. 10.1016/S2352-3018(19)30044-X 31060875

[29] Hu H Yan H Liu X Xu X Xu J Qiu T. Trends in late HIV diagnosis among men who have sex with men in Jiangsu province, China: results from four consecutive community-based surveys, 2011-2014. PLoS ONE. (2017) 12:e0172664. 10.1371/journal.pone.0172664 28278195 PMC5344382

[30] Moyer VA. U. S preventive services task force screening for HIV: US preventive services task force recommendation statement. Ann Intern Med. (2013) 159:51–60. 10.7326/0003-4819-159-1-201307020-00645 23698354

[31] Gullón A Verdejo J de Miguel R Gómez A Sanz J. Factors associated with late diagnosis of HIV infection and missed opportunities for earlier testing. AIDS Care. (2016) 28:1296–300. 10.1080/09540121.2016.1178700 27144427

[32] Conway AS Esteve A Fernández-Quevedo M Casabona J; PISCIS Study Group. Determinants and outcomes of late presentation of HIV infection in migrants in Catalonia, Spain: PISCIS cohort 2004-2016. J Immigr Minor Health. (2019) 21:920–30. 10.1007/s10903-018-0834-2 30377891

[33] WHO. Consolidated Guidelines on HIV Testing Services. Geneva, Switzerland. (2019) Available online at: https://www.who.int/publications/i/item/978-92-4-155058-1 (accessed August 8, 2022).

